# Expression profile of plasma microRNAs and their roles in diagnosis of mild to severe traumatic brain injury

**DOI:** 10.1371/journal.pone.0204051

**Published:** 2018-09-18

**Authors:** Xiaojing Qin, Lingzhi Li, Qi Lv, Qingming Shu, Yongliang Zhang, Yaping Wang

**Affiliations:** 1 Department of Histology and Embryology, Chongqing Medical University, Chongqing, China; 2 Department of Pathology, Affiliated Hospital of Logistic University of PAP, Tianjin, China; 3 Tianjin Key Laboratory for Prevention and Control of Occupational and Environmental Hazard, Tianjin, China; 4 Institute of Disaster Medicine, Tianjin University, Tianjin, China; 5 General Hospital of PAP, Beijing, China; University of Modena and Reggio Emilia, ITALY

## Abstract

Traumatic brain injury (TBI) is associated with trauma-related death. In this study, we evaluated differences in the expression of plasma microRNAs (miRNAs) in patients with different degrees of TBI, and explored the potential of miRNAs for use as diagnostic TBI biomarkers. The miRNA microarray results showed upregulation of 65, 33, and 16 miRNAs and downregulation of 29, 27, and 6 miRNAs in patients with mild, moderate, and severe TBI, respectively, compared with healthy controls. Thirteen miRNAs (seven upregulated and six downregulated) were found to be present in all TBI groups. Seven upregulated miRNAs were selected for validation in an enlarged cohort of samples and showed good diagnostic accuracy. The expression levels of miR-3195 and miR-328-5p were higher in the severe TBI group than in the mild and moderate TBI groups. In summary, our study demonstrates different expression profiles in plasma miRNAs among patients with mild to severe TBI. A subset of seven miRNAs can be used for diagnosis of TBI. Moreover, miR-3195 and miR-328-5p may be utilized during diagnosis to distinguish mild and moderate TBI from severe TBI.

## Introduction

Trauma is the fourth leading cause of death after heart disease, cancer, and cerebrovascular accidents. Traumatic brain injury (TBI), which accounts for most trauma-related deaths, is a leading cause of death in young people in China [1, 2]. There are 3–4 million TBI cases in China every year, and the mortality and disability rates occurring as a result of TBI are very high, in spite of the development of treatments [2].

The diagnosis of TBI is mainly based on a neurological examination of the patient, but also on imaging techniques such as computed tomography (CT) or magnetic resonance imaging (MRI) [3]. The Glasgow Coma Scale (GCS) is the most commonly used system for classifying TBI severity; a total score of 13–15 refers to mild TBI, 9–12 to moderate TBI, and 3–8 to severe TBI [4]. CT and MRI scans often fail to detect lesions caused by injury, due to limited sensitivity and the absence of micro-bleeds; this makes the detection of severe TBI easy, but the detection of mild and moderate TBI more difficult [5, 6]. Moreover, the GCS score has limitations in diagnosing mild TBI in the presence of polytrauma, alcohol abuse, use of sedatives, and psychological stress [6, 7].

Biomarkers have been utilized for the diagnosis of multiple diverse diseases and to uncover underlying pathologies in, for example, oncology, cardiology, hepatology, hematology, and infectious diseases [8–12]. Biomarkers are critical for the rational development of drugs and medical devices. However, there is significant confusion about the fundamental definitions and concepts involved in their use in research and clinical practice. A number of subtypes of biomarkers have been defined according to their putative applications. Recently, a joint task force from the U.S. Food and Drug Administration and the National Institutes of Health established common definitions, which divided biomarkers into a number of subtypes according to their putative applications. Among these subtypes, diagnostic biomarkers detect or confirm the presence of a disease or condition of interest, or identify an individual with a subtype of the disease [13, 14]. To date, the lack of diagnostic biomarkers in the field of TBI is a major barrier to the improvement of diagnostic evaluation and clinical care [15, 16]. To assist in the diagnosis of TBI, diagnostic biomarkers in the blood, urine, and cerebrospinal fluid (CSF) are under investigation. Up to now, the majority of TBI biomarker research has focused on protein profiling. The most widely studied blood biomarkers are S100β, glial fibrillary acidic protein, C-terminal hydrolase-L1, neuron-specific enolase, tau protein, amyloid β, and fatty acid binding proteins [17–19]. However, most biomarker candidates have not yet been tested in appropriate clinical settings.

MicroRNAs (miRNAs) represent a category of functional single-stranded small RNAs ranging from 17 to 25 nucleotides in length. MiRNAs mediate the translational control of target genes and play an important role in the regulation of physiological and pathological processes [20, 21]. In 2008, Mitchell et al. found that miRNAs can exist in a stable form in the plasma, escaping ribonuclease degradation [22]. Using high-throughput detection techniques, miRNA profiles are specific to various physiological and pathological conditions, wherein lies their potential as diagnostic biomarkers [23]. MiRNAs have been reported to be specific and sensitive biomarkers in many central nervous system diseases [24]. In this study, we first investigated plasma miRNA expression profiles by using Agilent Human miRNA microarray techniques (miRBase V21.0). The expression profiles of a total of 2549 miRNAs were determined in five patients with mild TBI, five with moderate TBI, and five with severe TBI, within 24 hours of injury, as well as in five healthy volunteers (HVs). Next, some candidate biomarkers were selected for validation using a quantitative reverse transcription polymerase chain reaction (RT-qPCR) technique in an enlarged, separate and independent cohort with 25 subjects in each group, including patients with different degrees of TBI, as well as healthy volunteers. A receiver operating characteristic (ROC) curve was generated to evaluate the accuracy of these candidate miRNAs in diagnosing TBI.

## Materials and methods

### Participants

Patients were recruited from the Department of Neurosurgery, Affiliated Hospital of Logistics University of PAP, between March 2016 and March 2017. The inclusion criteria were as follows: 1) aged between 18 and 60 years, 2) diagnosed with TBI, and 3) injury had occurred within 24 hours of arrival at the emergency department. The exclusion criteria were as follows: 1) pregnant or nursing; 2) significant polytrauma that would interfere with follow-up and outcome assessment; 3) uncontrolled blood pressure and additional cardiovascular disease; 4) major substance abuse or alcoholism; and 5) major debilitating neurological diseases. Mild TBI included patients with a non-penetrating head trauma and GCS score ≥13, moderate TBI included those with a GCS score of 9–12, and severe TBI included those with a GCS score ≤8. This prospective cohort study was approved by the Logistics University of PAP Review Board prior to data collection. Written informed consent was obtained from all human subjects or from their legal authorized representatives prior to enrollment. All experiments were performed in accordance with relevant guidelines and approval from the Logistics University of PAP Review Board.

### Plasma preparation and RNA isolation

The blood samples in ethylene diamine tetra-acetic acid anticoagulant tubes were processed for plasma isolation within 2 h of being drawn. Whole blood was centrifuged at 3000 rpm (1408 rcf) for 10 min at room temperature. Plasma was divided into aliquots and stored at -80°C.

Plasma RNA isolation was performed using the mirVanaTM PARISTM Serum/Plasma Kit (Ambion, Austin, TX, USA). The RNA quantity and quality were assessed using a NanoDrop ND-1000 (Thermo Fisher, Boston, MA, USA) and Agilent 2100 Bioanalyzer (Agilent Technologies, Santa Clara, CA, USA). We used 400 μL and 200 μL of plasma for RNA isolation in the miRNA microarray assay and the RT-qPCR validation experiment, respectively.

### miRNA microarray analysis

Human miRNA microarrays from Agilent Technologies (Agilent SurePrint Human miRNA v21.0 microarray, G4872A) (Agilent Technologies, Santa Clara, CA, USA) were used in plasma samples. The total RNA (100 ng) derived from plasma samples was labelled with Cy3. The labelling and hybridization were performed according to the protocols in the Agilent miRNA microarray system. After hybridization, slides were scanned with the Agilent Microarray Scanner and raw data were normalized using the quantile algorithm in GeneSpring software 12.6.

### Analysis of microarray data

Normalized data were obtained using the Quantile algorithm in the GeneSpring software program (version 11.0, Agilent Technologies), and these data were transformed to base 2. A relative fold change >2 in the differential expression of miRNAs and a *P*<0.05 were considered significant. The Gene Cluster (version 3.0) and Java Treeview software programs were used to perform the hierarchical cluster analysis of differentially expressed miRNAs and to visualize the miRNAs.

### Quantitative reverse transcription polymerase chain reaction

For the testing of the candidate miRNAs acquired on microarrays, RT-qPCR was performed. The total RNA was reverse transcribed using the miRcute Plus miRNA First-Strand cDNA Synthesis Kit (Tiangen Biotech, Beijing, China) on a Proflex Base PCR System (Thermo Fisher Scientific, MA, USA). Real-time PCR reactions were performed on the PikoReal 96 (Thermo Scientific, MA, USA) in a 20-μL reaction volume containing 2 μL reverse transcription product, 10 μL 2×miRcute Plus miRNA Premix (with SYBR & ROX) (Tiangen Biotech), 0.4 μL PCR forward primer (5 μM, Tiangen Biotech), 0.4 μL reverse primer (10 μM, Tiangen Biotech), and 7.2 μL ddH_2_O. The reactions were incubated at 95°C for 15 min, followed by 40 cycles (94°C for 20 s and 60°C for 34 s). For the exogenous control, wascel-miR-39 was used; 1 μL of cel-miR-39 mimic (100 nM) was added into 200-μL plasma samples before RNA isolation. Each miRNA was calibrated against tocel-miR-39 to get a delta Ct (ΔCt) value for each miRNA (miRNA Ct value−cel-miR-39 Ct value). 2^-ΔCt^ values in each group were used for the following statistical analysis. Expression fold changes were calculated busing the 2^−ΔΔCt^ method.

### miRNA target computational analysis

The target genes of differentially expressed miRNAs were predicted by the following three prediction databases: TargetScan (http://www.targetscan.org), miRanda (http://www.microrna.org/microrna/home.do), and PicTar (http://pictar.mdc-berlin.de/). The enrichment *P*-values of both the gene ontology (GO) analysis and the Kyoto Encyclopedia of Genes and Genomes (KEGG) pathway enrichment analysis were calculated using Fisher’s exact test, corrected by enrichment q-values (the false discovery rate) that were calculated using John Storey's method. The GO terms were identified in the biological process, cellular component, and molecular function categories. The KEGG database (http://www.genome.jp/kegg/tool/search_pathway.html) was used to map the predicted targets of the miRNAs.

### Statistical analysis

Data are presented as mean ± standard deviation (SD). For the miRNA microarrays, the differences in miRNA abundance between HVs and TBI samples (background-adjusted, log2-transformed, balanced fluorescence values) as detected by microarray analysis were calculated, and changes between the signal intensities were evaluated using Student’s t-tests. For the RT-qRCR, 2^-ΔCt^ values obtained from the RT-qPCR experiments in each group were used for the statistical analysis [25]. Data were compared using one-way analysis of variance (ANOVA) and Tukey’s post-hoc test, or an independent samples-test comparison between the HV, mild TBI, moderate TBI, and severe TBI groups. An ROC analysis was utilized to calculate sensitivity and specificity of each biomarker. All analyses were carried out on SPSS version 19.0, with *P*<0.05 considered statistically significant.

## Results

### Patient characteristics

The characteristics of the study participants are presented in Table 1. A total of 90 patients with TBI and 30 HVs were enrolled in this study. In the microarray groups, the miRNA profiles of the plasma samples from five subjects in each group were screened using a miRNA microassay. The sampling times in the mild, moderate, and severe TBI groups were 7.54±2.81, 6.08±0.79, and 6.16±1.19, respectively. There were no statistical differences between the groups. In the validation groups, separate and independent cohorts of 25 subjects in each group were enrolled for the validation of selected miRNAs. The sampling times in the mild, moderate, and severe TBI groups were 7.15±1.54, 7.38±1.60, and 6.60±1.63, respectively. There were no statistical differences between the groups.

**Table 1:** Clinical characteristics of the study subjects.

	Study group	Number of samples	Age [Mean±SD, (Range)]	Gender (M/F)	Sampling time (hours)	GCS
Microarray group	HV	5	45.2±8.9 (33–55)	4/1		
	Mild TBI	5	41.4±9.3 (27–50)	4/1	7.54±2.81	13.6±0.5 (13–14)
	Moderate TBI	5	43.6±6.7 (35–50)	3/2	6.08±0.79	10.0±1.0 (9–11)
	Severe TBI	5	48.6±9.8 (32–52)	4/1	6.16±1.19	4.4±1.5 (3–6)
Validation group	HV	25	39.7±9.3 (29–59)	18/7		
	Mild TBI	25	44.9±10.9(24–60)	16/9	7.15±1.54	13.7±0.5 (13–14)
	Moderate TBI	25	46.0±8.8(27–60)	19/6	7.38±1.60	10.6±1.0(9–12)
	Severe TBI	25	45.4±8.0 (32–57)	17/8	6.60±1.63	5.4±1.6(3–8)

### miRNA expression profiles

**Fig 1:**
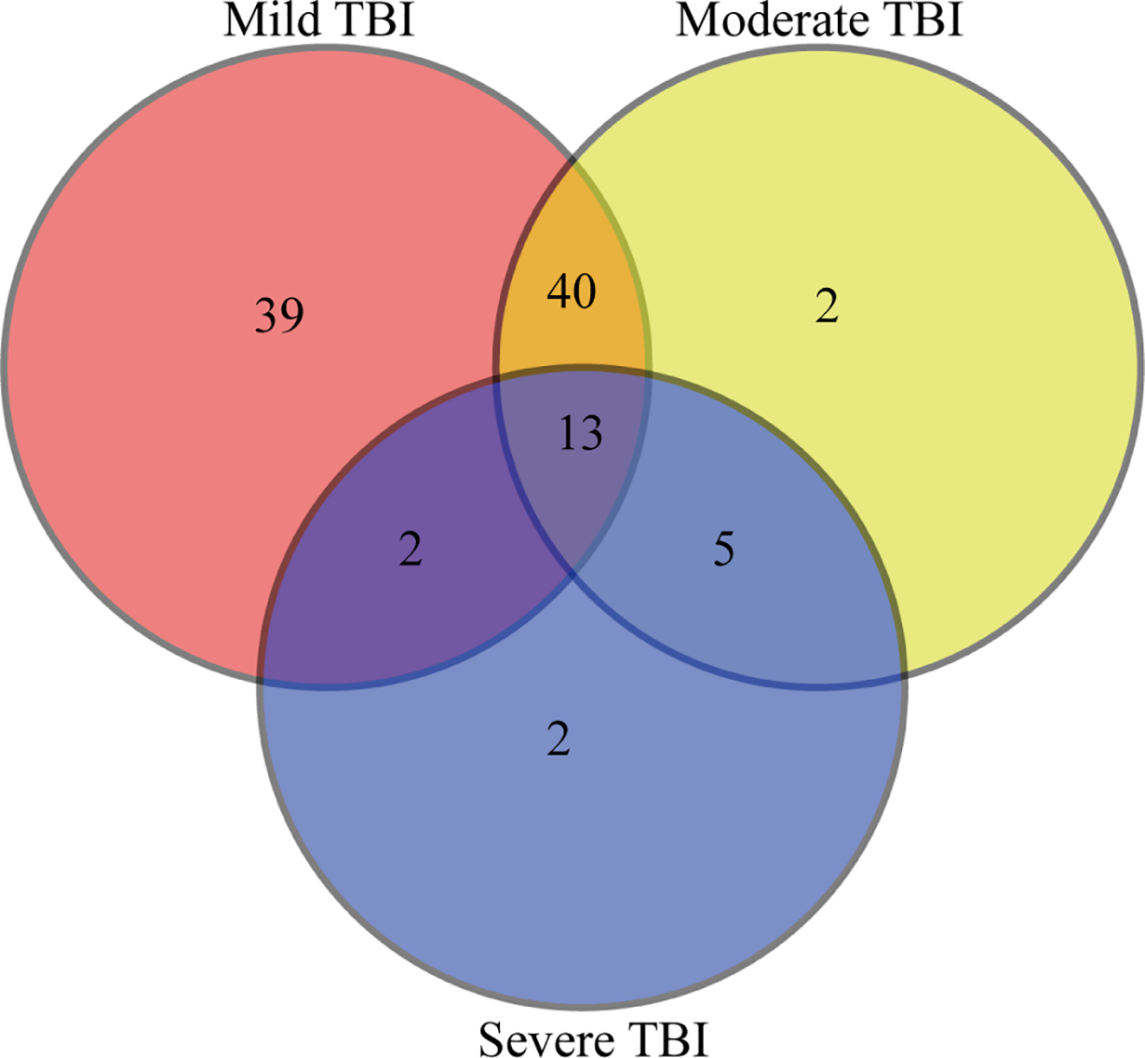
MiRNA expression pattern. Venn diagram shows overlapping different miRNAs in mild, moderate and severe TBI in comparison to health volunteers.

**Fig 2:**
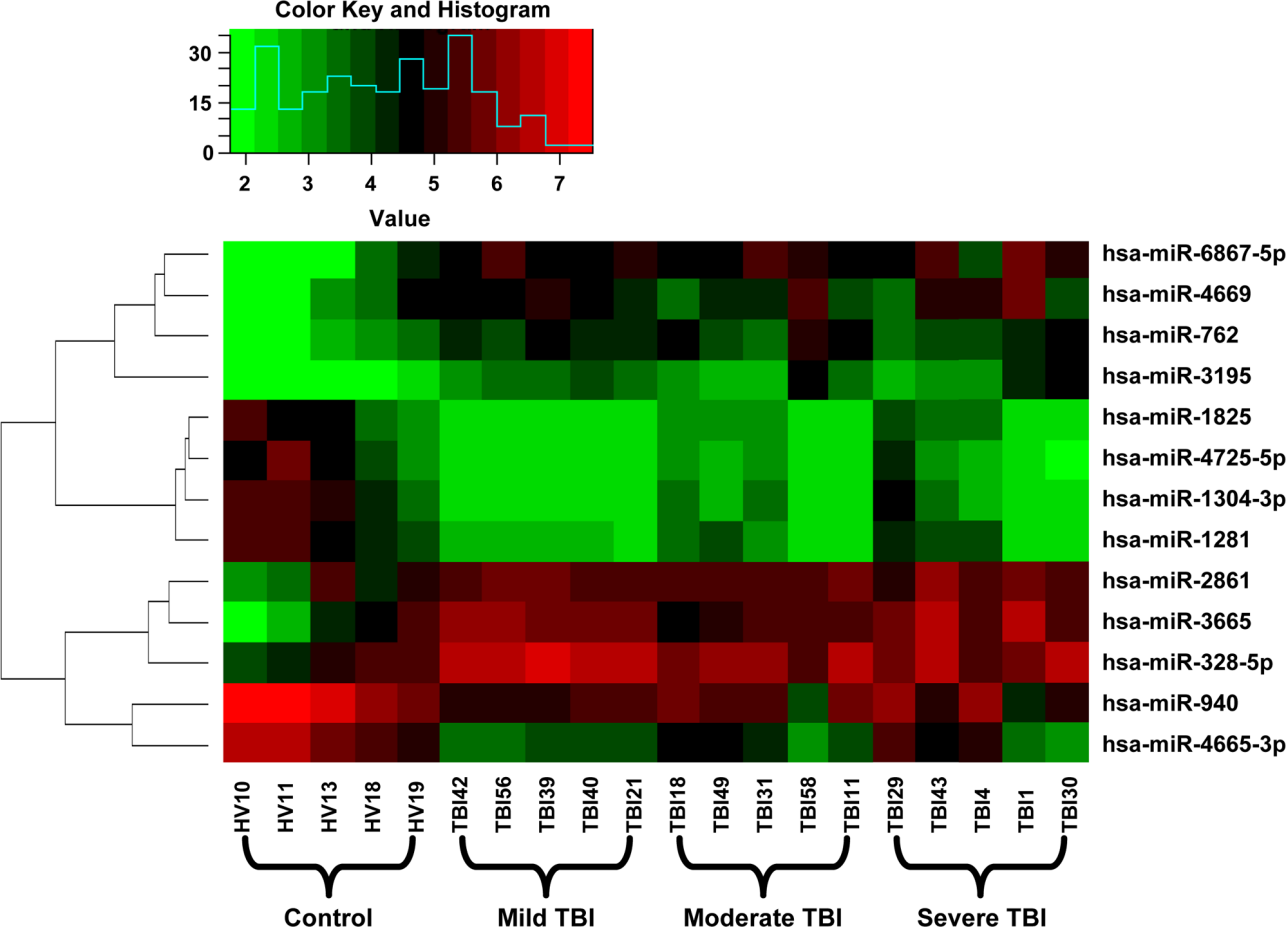
Hierarchical clustering of 13 common miRNAs altered in all TBI groups. The Hierarchical clustering of hsa-miR-1281, hsa-miR-1304-3p, hsa-miR-1825, hsa-miR-2861, hsa-miR-3195, hsa-miR-328-5p, hsa-miR-3665, hsa-miR-4665-3p, hsa-miR-4669, hsa-miR-4725-5p, hsa-miR-6867-5p, hsa-miR-762 and hsa-miR-940 were conducted. The red and green colors in the heat map represent miRNA expression levels, respectively.

A microarray containing probes of 2549 human miRNAs was initially used to screen the significant differential expression levels of miRNAs between the groups with different degrees of TBI and the control group. After the normalization, the fold change for the plasma miRNAs in the mild, moderate, and severe TBI groups was calculated using healthy control subjects as the baseline. Compared with the healthy control group, 65, 33, and 16 miRNAs were upregulated, and 29, 27, and six miRNAs were downregulated in patients with mild, moderate, and severe TBI, respectively (S1–S3 Tables). Among these, 13 miRNAs (seven upregulated and six downregulated) were found to be present in all TBI groups (fold change >2, *P*<0.05) (Table 2, Fig 1). We also performed hierarchical clustering on the normalized data to understand the expression of the 13 common miRNAs between the groups. A clustering analysis showed segregated data suggesting different expressions of these 13 miRNAs across the healthy control and the TBI groups (Fig 2).

**Table 2:** Common miRNAs altered in plasma samples of mild, moderate and severe TBI patients.

No.	miRNA	Mild vs control		Moderate vs control		Severe vs control	
		Fold change	p-value	Fold change	p-value	Fold change	p-value
1	hsa-miR-6867-5p	3.28	1.36E-02	4.05	7.41E-03	4.32	6.29E-03
2	hsa-miR-3665	3.24	2.58E-02	2.66	4.26E-02	3.56	2.14E-02
3	hsa-miR-328-5p	2.89	5.32E-03	2.30	1.85E-02	2.33	1.63E-02
4	hsa-miR-762	2.72	6.99E-03	2.96	5.45E-03	2.39	1.22E-02
5	hsa-miR-3195	2.54	1.24E-03	3.17	1.02E-02	3.23	6.05E-03
6	hsa-miR-4669	2.40	3.23E-02	2.40	4.95E-02	2.75	4.13E-02
7	hsa-miR-2861	2.19	2.83E-02	2.07	3.44E-02	2.13	3.23E-02
8	hsa-miR-940	0.32	7.81E-03	0.32	1.01E-02	0.37	1.99E-02
9	hsa-miR-1281	0.25	1.06E-03	0.24	9.00E-04	0.39	2.36E-02
10	hsa-miR-1825	0.25	7.70E-03	0.29	1.11E-02	0.40	4.69E-02
11	hsa-miR-4665-3p	0.22	1.24E-03	0.21	9.69E-04	0.33	1.54E-02
12	hsa-miR-4725-5p	0.21	7.46E-03	0.26	1.24E-02	0.37	4.06E-02
13	hsa-miR-1304-3p	0.18	2.34E-03	0.26	5.00E-03	0.38	3.29E-02

### miRNA targets and gene ontology, pathway analysis of different miRNAs in different degree TBI groups

To understand the physiopathological roles of the different distributions of miRNAs in the plasma of patients with mild to severe TBI, GO and pathway enrichment analyses of predicted miRNA targets were conducted. We compared the top 15 biological processes of the GO analysis of miRNA targets in different TBI groups; the results showed that 10 biological processes were present in both the mild and moderate TBI group, which indicates similar physiopathological phenotypes. Different from these two TBI groups, only one biological process, the regulation of systemic arterial blood pressure by vasopressin, was present in the severe TBI group as well as the mild and moderate TBI groups. The other top 15 biological processes observed in the severe TBI group included synaptic vesicle clustering, somatic motor neuron differentiation, presynaptic active zone assembly, optic nerve morphogenesis, and noradrenergic neuron differentiation. These processes were more directly associated with neuron activity (Table 3).

**Table 3:** Gene ontology enrichment analysis of miRNA targets in different degree TBI groups (Top 15 processes were showed).

No.	Mild	Moderate	Severe
1	somite specification	selenocysteine incorporation	synaptic vesicle clustering
2	selenocysteine incorporation	response to tumor cell	somatic motor neuron differentiation
3	ribosomal subunit export from nucleus	renin secretion into blood stream	regulation of systemic arterial blood pressure by vasopressin
4	ribosomal small subunit assembly	regulation of systemic arterial blood pressure by vasopressin	regulation of skeletal muscle contraction by calcium ion signaling
5	renin secretion into blood stream	regulation of systemic arterial blood pressure by carotid sinus baroreceptor feedback	regulation of protein heterodimerization activity
6	regulation of systemic arterial blood pressure by vasopressin	regulation of response to tumor cell	regulation of protein deubiquitination
7	regulation of systemic arterial blood pressure by carotid sinus baroreceptor feedback	positive regulation of receptor recycling	purine ribonucleosidebisphosphate biosynthetic process
8	regulation of glutamine family amino acid metabolic process	regulation of immune response to tumor cell	protein repair
9	regulation of germinal center formation	regulation of germinal center formation	presynaptic active zone assembly
10	regulation of dendritic cell cytokine production	regulation of dendritic cell cytokine production	positive thymic T cell selection
11	positive regulation of tolerance induction	positive regulation of tolerance induction	positive regulation of viral entry into host cell
12	positive regulation of receptor recycling	positive regulation of response to tumor cell	positive regulation of toll-like receptor 3 signaling pathway
13	positive regulation of neurotransmitter secretion	positive regulation of receptor recycling	Pointed-end actin filament capping
14	positive regulation of cytokine secretion involved in immune response	positive regulation of cytokine secretion involved in immune response	optic nerve morphogenesis
15	positive regulation of T cell tolerance induction	positive regulation of T cell tolerance induction	noradrenergic neuron differentiation

The KEGG pathway is a service offered by the Kyoto Encyclopedia of Genes and Genomes, by constructing manually curated pathway maps that represent the current knowledge on biological networks in graph models [26]. A pathway enrichment analysis suggested that several pathways were present in all TBI groups, including the p53 signaling pathway, the mTOR signaling pathway, the TGF-β signaling pathway, SNARE interactions in vesicular transport, the nicotinate and nicotinamide metabolism, and the neurotrophin signaling pathway. Moreover, only two neurobiological pathways, pantothenate and CoA biosynthesis and long-term depression, were present in the top 15 pathways of the TBI groups (Table 4).

**Table 4:** KEGG pathway enrichment analysis of miRNA targets in different degree TBI groups (Top 15 pathways were showed).

No.	Mild	Moderate	Severe
1	p53 signaling pathway	p53 signaling pathway	p53 signaling pathway
2	mTOR signaling pathway	mTOR signaling pathway	mTOR signaling pathway
3	mRNA surveillance pathway	TGF-beta signaling pathway	Thyroid cancer
4	TGF-beta signaling pathway	SNARE interactions in vesicular transport	TGF-beta signaling pathway
5	SNARE interactions in vesicular transport	Purine metabolism	Shigellosis
6	Pancreatic cancer	Phosphatidylinositol signaling system	SNARE interactions in vesicular transport
7	Pantothenate and CoA biosynthesis	Non-small cell lung cancer	Purine metabolism
8	Pancreatic cancer	Nicotinate and nicotinamide metabolism	Prostate cancer
9	Osteoclast differentiation	Neurotrophin signaling pathway	Phosphatidylinositol signaling system
10	One carbon pool by folate	Melanoma	Pancreatic cancer
11	Non-small cell lung cancer	Melanogenesis	Non-small cell lung cancer
12	Nicotinate and nicotinamide metabolism	Inositol phosphate metabolism	Nicotinate and nicotinamide metabolism
13	Neurotrophin signaling pathway	Hypertrophic cardiomyopathy (HCM)	Neurotrophin signaling pathway
14	Melanoma	Hedgehog signaling pathway	NOD-like receptor signaling pathway
15	Long-term depression	Glycosphingolipid biosynthesis—lacto and neolacto series	Melanoma

### Validation of candidate miRNAs

**Fig 3:**
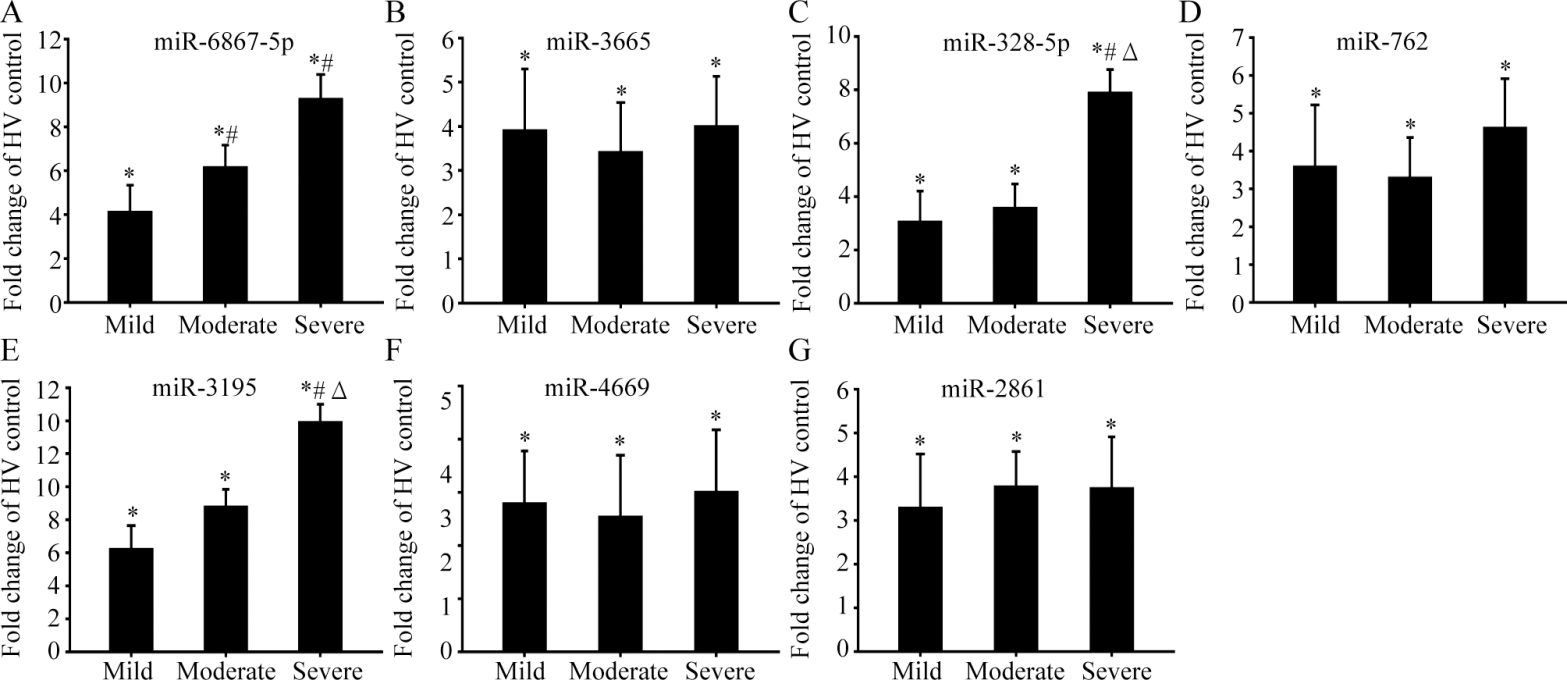
Validation of candidate miRNAs by real time PCR. A-G, The expression levels of 7 candidate miRNAs (miR-6867-5p, miR-3665, miR-328-5p, miR-762, miR-3195, miR-4669 and miR-2861) were validated by using real time PCR in aseparate and independentcohort of 25subjects in each group with different degree TBI patients and healthy volunteers. *represents compared with HV group, *P*<0.05; #represents compared with mild TBI group, *P*<0.05; Δ represents compared with moderate TBI group.

Next, we selected seven candidate miRNAs—miR-6867-5p, miR-3665, miR-328-5p, miR-762, miR-3195, miR-4669, and miR-2861—to validate their expressions using RT-qPCR. The microarray results showed that the plasma levels of these miRNAs were upregulated in all TBI groups (Table 2). A total of 100 separate and independent samples (25 in each group) were available for RT-qPCR analysis. We used cel-miR-39 as an exogenous control. The validation results showed significant upregulation of the miRNAs in all three TBI groups when compared to the HV samples, which is consistent with our miRNA microarray results (Fig 3). Moreover, the expression levels of miR-3195 and miR-328-5p were higher in the severe TBI group than in the mild and moderate TBI groups (Fig 3C & 3E). The expression levels of miR-6867-5p in the moderate and severe TBI groups were higher than those in the mild TBI group (Fig 3A).

### Diagnostic performance of miRNA candidates

**Fig 4:**
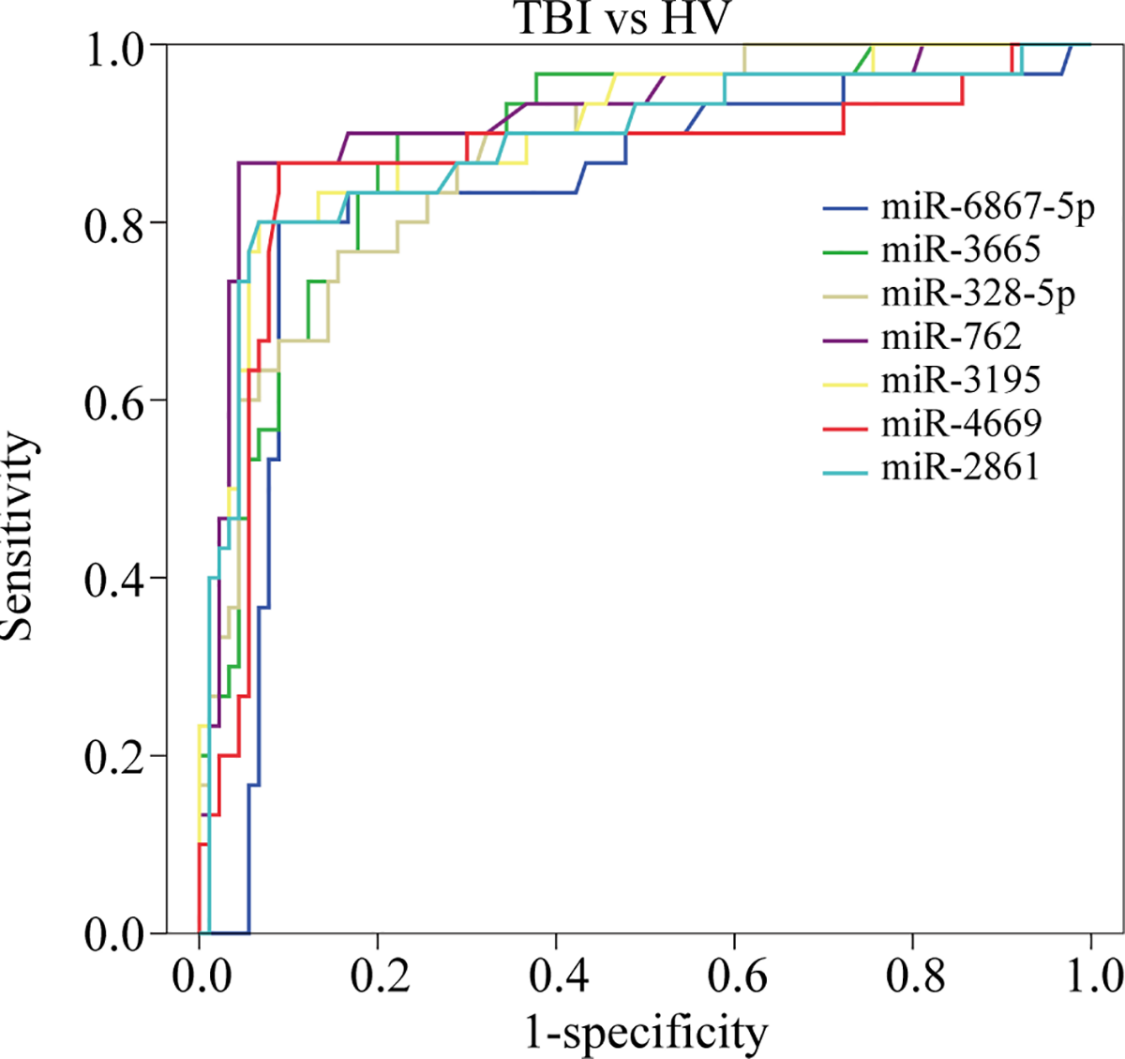
ROC curve analysis evaluates the diagnostic performance of the selected microRNA panels for distinguishing TBI from controls. The Area Under roc Curve (AUC) values were as following: miR-6867-5p miR-6867-5p (0.854, *P*<0.001), miR-3665 (0.877, *P*<0.001), miR-328-5p (0.888, *P*<0.001), miR-762 (0.916, *P*<0.001), miR-3195 (0.899, *P*<0.001), miR-4669 (0.907, *P*<0.001), and miR-2861 (0.913, *P*<0.001).

Next, we evaluated the accuracy of the seven common upregulated miRNAs in diagnosing TBI. An ROC analysis was conducted to calculate the area under the curve (AUC) to identify the diagnostic accuracy of the miRNAs. The analysis revealed the following AUC values: miR-6867-5p (0.854, *P*<0.001), miR-3665 (0.877, *P*<0.001), miR-328-5p (0.888, *P*<0.001), miR-762 (0.916, *P*<0.001), miR-3195 (0.899, *P*<0.001), miR-4669 (0.907, *P*<0.001), and miR-2861 (0.913, *P*<0.001) (Fig 4). All miRNAs thus showed good diagnostic accuracy.

### Determination of diagnostic potential for candidate miRNAs for mild TBI

**Fig 5:**
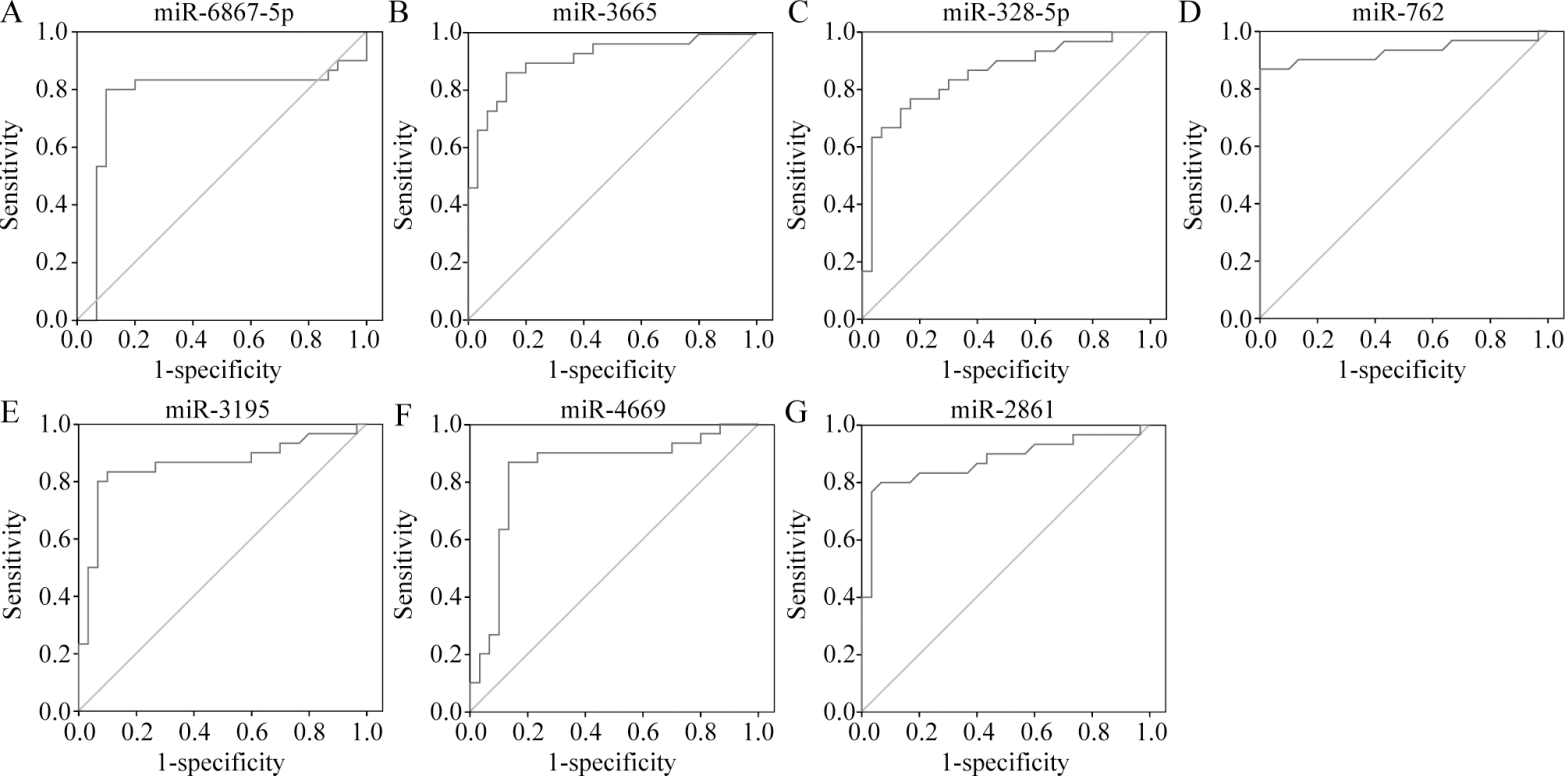
ROC curve analysis evaluates the diagnostic performance of the selected microRNA panels for distinguishing mild TBI from controls. A-G, The AUC values were as following: miR-6867-5p (0.765, *P*<0.001), miR-3665 (0.916, *P*<0.001), miR-328-5p (0.855, *P*<0.001), miR-762 (0.921, *P*<0.001), miR-3195 (0.859, *P*<0.001), miR-4669 (0.894, *P*<0.001), and miR-2861 (0.898, *P*<0.001).

Since the ROC curve results indicated that candidate miRNAs have a good diagnostic potential for TBI, we next evaluated if these candidate miRNAs could be used to diagnose mild TBI. The analysis yielded the following AUC values: miR-6867-5p (0.765, *P*<0.001), miR-3665 (0.916, *P*<0.001), miR-328-5p (0.855, *P*<0.001), miR-762 (0.921, *P*<0.001), miR-3195 (0.859, *P*<0.001), miR-4669 (0.894, *P*<0.001), and miR-2861 (0.898, *P*<0.001) (Fig 5A–5G). Thus, while these miRNAs show fair diagnostic accuracy in identifying patients with mild TBI overall, miRNA-3665 and miR-762 show higher accuracy for diagnosing this group of patients.

## Discussion

miRNAs are well-conserved in both plants and animals and are thought to be a vital and evolutionarily ancient component of gene regulation [27]. The human genome may encode over 2000 miRNAs, which are abundant in many mammalian cell types and appear to target more than 60% of the genes in humans and other mammals [27, 28]. Dysregulation of miRNAs has been shown to be associated with multiple diseases, such as cancer [29], heart disease [30], kidney disease [31], diabetes [32], nervous system diseases [33, 34], and other diseases. However, little research has focused on the roles of miRNAs in TBI. In this study, we evaluated the changes in circulating miRNA expression profiles in the plasma of patients with mild to severe TBI, using miRNA microarrays. The microarray analysis identified 94 miRNAs (65 upregulated, 29 downregulated) in mild TBI, 60 miRNAs (33 upregulated, 27 downregulated) in moderate TBI, and 22 miRNAs (16 upregulated, six downregulated) in severe TBI. Thirteen miRNAs (seven upregulated and six downregulated) were found to be present in all TBI groups.

Next, we focused on the common upregulated miRNAs in all (mild to severe) TBI groups, compared with the healthy control group, validated the changes by real-time PCR in an enlarged, separate, and independent cohort of 25 subjects in each group, and evaluated the diagnostic value of these miRNAs for TBI. The validation assay confirmed that the seven candidate miRNAs were upregulated in all TBI groups. A ROC curve analysis demonstrated that all seven miRNAs showed good diagnostic accuracy for distinguishing patients with TBI from healthy controls. Furthermore, the expression levels of hsa-miR-3195 and hsa-miR-328-5p were higher in the severe TBI group than in the mild and moderate groups, which indicates that these two miRNAs may also be utilized during diagnosis to distinguish mild and moderate TBI from severe TBI.

Sample quality is an important factor as many dysregulated miRNAs found in this study can be affected by factors such as hemolysis or platelet contamination. Numerous studies have shown that cellular contamination and hemolysis of plasma (and serum) samples can be a major cause of variation in miRNA levels not related to any biological difference [35, 36]. In the case of serum samples, clot formation can result in cell lysis and the lease of miRNA within blood cells and platelets. In the case of plasma samples, cells can be inadvertently aspirated during collection [37]. Thus, in this study, we focused on the alterations in miRNA expression profiles in the plasma samples of patients with TBI. To ensure the plasma sample quality, the samples were discarded if there was turbidity, precipitation, or hemolysis. Moreover, we did not aspirate about 50 μL of plasma after centrifugation, to avoid contamination of the cells.

To date, several studies have reported on different miRNA expression after TBI. Some altered miRNAs found in our study showed an overlap with these reports. Bhomia et al. evaluated the serum miRNA expression profile in patients with mild to severe TBI by using TaqMan real-time PCR, and then validated candidate biomarkers. They showed that 10 miRNAs were present in both the mild and moderate as well as the severe TBI group. Among these miRNAs, both our study and Bhomia et al.’s study found upregulation of hsa-miR-328 in all TBI groups. An advanced validation assay showed higher expressions of hsa-miR-328 in the severe TBI group than in the mild and moderate TBI groups. Moreover, five upregulated miRNAs in mild TBI—hsa-miR-20a-5, hsa-miR-27a-3p, hsa-miR-30d-5p, hsa-miR-451a, and hsa-miR-92a-3p were found in both our study and Bhomia et al.’s study [38]. Another study also tested the upregulation of the plasma levels of hsa-miR-92a within the first 24 h after TBI injury [39]. Interestingly, Pietro and colleagues evaluated serum miRNA expression profiles in patients with severe TBI by using the TaqMan low-density array, and then validated candidate biomarkers for mild and severe TBI. They found that hsa-miR-425-5p was significantly downregulated in mild TBI at 0–12 h and returned to normal levels after 48 h [7]. However, our microarray results differed from these findings in that they showed that hsa-miR-425-5p was upregulated in mild TBI (S1 Table). Further validation studies must be conducted to verify the dynamic changes of this miRNA in TBI.

It has been demonstrated that circulating miRNAs can be enwrapped with exosomes or microvesicles and transferred from one cell to another through the exchange of exosomes [22, 40]. These vesicle-enwrapped circulating miRNAs may function as an intercellular communication system in the body [41]. We explored the underlying biological function of changed plasma miRNAs in patients with different degrees of TBI. A GO analysis of miRNA targets showed that only one biological process, the regulation of systemic arterial blood pressure by vasopressin, was present in the top 15 biological processes of all three TBI groups. It has been reported that systemic hypotension can compromise cerebral hemodynamics and cause cerebral ischemia after TBI [42]; lower mean arterial blood pressure has been considered an independent prognostic factor in patients with TBI [43]. Guidelines provided by the Brain Trauma Foundation for the management of TBI recommend avoiding hypotension by maintaining cerebral perfusion pressure [44]. In addition to the regulation of systemic arterial blood pressure by vasopressin, there were two biological processes—renin secretion into the bloodstream and regulation of systemic arterial blood pressure by carotid sinus baroreceptor feedback—in the top 15 biological processes of the mild and moderate TBI groups. This indicates that miRNAs may participate in the regulation of systemic arterial blood pressure after TBI.

More evidence supports that immune system activation and inflammation are central mediators of secondary injury after brain trauma [45]. TBI often promotes the disruption of the blood-brain barrier, thereby induces damage-associated molecular patterns to be released into both the CSF and serum, and triggers the inflammatory response after TBI [45, 46]. Our study identifies four inflammation-related processes in the top 15 biological processes of the mild and moderate TBI groups, including positive regulation of the induction of tolerance, regulation of dendritic cell cytokine production, positive regulation of cytokine secretions involved in the immune response, and positive regulation of the induction of T cell tolerance. However, two different inflammation-related processes—positive regulation of the toll-like receptor 3 signaling pathway, and positive thymic T cell selection—were present in the top 15 biological processes of the severe TBI groups. These results indicate that inflammatory responses differ between patients with mild, moderate, and severe TBI. Moreover, the GO analysis of miRNA target genes showed that five neurobiological processes were present in the top 15 biological processes of the severe TBI group that were positively correlated with the severity of TBI in these patients.

The KEGG pathway enrichment analysis showed that six pathways were present in all TBI groups (Table 4). Among these pathways, the p53 signaling pathway, mTOR signaling pathway, and TGF-β signaling pathway have been reported to be activated in TBI and involved in inflammation and apoptosis [47–49]. Inhibition of these three pathways showed a neuroprotective effect in TBI [49–51]. SNARE proteins are a large protein superfamily that mediates the docking of synaptic vesicles with the presynaptic membrane in neurons. Impaired SNARE complex formation was reported after TBI [52]. Goffus et al. showed that sustained delivery of nicotinamide could limit cortical injury and improve functional recovery following TBI [53]. Neurotrophins, including BDNF, NT-3, NT-4, and NGF, play an active role in the development, maintenance, and survival of the cells of the central nervous system. Several studies support that neurotrophins could protect hippocampal neurons and restore neural connectivity following TBI [54, 55]. The pathway enrichment analysis results thus demonstrate that the changes in serum miRNAs may participate in the process of TBI by regulating these pathways.

In summary, a cohort of seven plasma miRNAs identified in our study can be used as acute biomarkers of TBI. Two miRNAs in particular, miR-3195 and miR-328-5p, may be utilized during diagnosis to distinguish mild and moderate TBI from severe TBI. Further studies will be needed to identify the functions and roles of these candidate miRNAs in TBI.

## Supporting information

S1 Table MiRNAs altered in plasma samples of mild TBI compared with HV. (DOCX)

S2 Table MiRNAs altered in plasma samples of moderate TBI compared with HV. (DOCX)

S3 Table MiRNAs altered in plasma samples of severe TBI compared with HV. (DOCX)
