# Supplementary material for: Expression profile of plasma microRNAs and their roles in diagnosis of mild to severe traumatic brain injury
Source: PLoS One. 2018 Sep 18;13(9):e0204051. doi: 10.1371/journal.pone.0204051 (PMC6143266; doi:10.1371/journal.pone.0204051)
Supplement: S1 Table — (DOCX) [file pone.0204051.s001.docx]

**Table S1** MiRNAs altered in plasma samples of Mild TBI compared with HV.

| **No.** | **miRNA** | **Mature sequence** | **Fold change** | **p-value** |
| --- | --- | --- | --- | --- |
| 1 | hsa-miR-24-3p | uggcucaguucagcaggaacag | 5.7 | 0.00 |
| 2 | hsa-miR-130a-3p | cagugcaauguuaaaagggcau | 5.61 | 0.00 |
| 3 | hsa-miR-223-3p | ugucaguuugucaaauacccca | 5.49 | 0.01 |
| 4 | hsa-miR-29c-3p | uagcaccauuugaaaucgguua | 5.16 | 0.00 |
| 5 | hsa-miR-27a-3p | uucacaguggcuaaguuccgc | 4.15 | 0.00 |
| 6 | hsa-miR-3141 | gagggcggguggaggagga | 4.07 | 0.00 |
| 7 | hsa-miR-4800-5p | aguggaccgaggaaggaagga | 4.01 | 0.00 |
| 8 | hsa-miR-6727-5p | cucggggcaggcggcugggagcg | 3.85 | 0.01 |
| 9 | hsa-miR-6831-5p | uagguagagugugaggaggagguc | 3.8 | 0.00 |
| 10 | hsa-miR-142-5p | cauaaaguagaaagcacuacu | 3.76 | 0.00 |
| 11 | hsa-miR-30e-5p | uguaaacauccuugacuggaag | 3.74 | 0.00 |
| 12 | hsa-miR-3610 | gaaucggaaaggaggcgccg | 3.73 | 0.00 |
| 13 | hsa-miR-3138 | uguggacagugagguagagggagu | 3.72 | 0.00 |
| 14 | hsa-miR-4499 | aagacugagaggaggga | 3.58 | 0.00 |
| 15 | hsa-miR-106b-5p | uaaagugcugacagugcagau | 3.54 | 0.02 |
| 16 | hsa-miR-101-3p | uacaguacugugauaacugaa | 3.53 | 0.00 |
| 17 | hsa-miR-20a-5p | uaaagugcuuauagugcagguag | 3.52 | 0.01 |
| 18 | hsa-miR-4778-5p | aauucuguaaaggaagaagagg | 3.44 | 0.01 |
| 19 | hsa-miR-4634 | cggcgcgaccggcccgggg | 3.37 | 0.01 |
| 20 | hsa-miR-6867-5p | uguguguguagaggaagaaggga | 3.28 | 0.01 |
| 21 | hsa-miR-3665 | agcaggugcggggcggcg | 3.24 | 0.03 |
| 22 | hsa-miR-134-5p | ugugacugguugaccagagggg | 3.12 | 0.00 |
| 23 | hsa-miR-17-5p | caaagugcuuacagugcagguag | 3.08 | 0.03 |
| 24 | hsa-miR-4298 | cugggacaggaggaggaggcag | 3.02 | 0.00 |
| 25 | hsa-miR-185-5p | uggagagaaaggcaguuccuga | 3.01 | 0.01 |
| 26 | hsa-miR-6833-5p | guguggaagaugggaggagaaa | 2.92 | 0.00 |
| 27 | hsa-miR-140-3p | uaccacaggguagaaccacgg | 2.91 | 0.02 |
| 28 | hsa-miR-328-5p | gggggggcaggaggggcucaggg | 2.89 | 0.01 |
| 29 | hsa-miR-107 | agcagcauuguacagggcuauca | 2.86 | 0.01 |
| 30 | hsa-miR-762 | ggggcuggggccggggccgagc | 2.72 | 0.01 |
| 31 | hsa-miR-425-5p | aaugacacgaucacucccguuga | 2.7 | 0.00 |
| 32 | hsa-miR-93-5p | caaagugcuguucgugcagguag | 2.7 | 0.01 |
| 33 | hsa-miR-6728-5p | uugggaugguaggaccagagggg | 2.69 | 0.00 |
| 34 | hsa-miR-4739 | aagggaggaggagcggaggggcccu | 2.66 | 0.01 |
| 35 | hsa-miR-451a | aaaccguuaccauuacugaguu | 2.64 | 0.05 |
| 36 | hsa-miR-6812-5p | auggggugagauggggaggagcagc | 2.59 | 0.00 |
| 37 | hsa-miR-26a-5p | uucaaguaauccaggauaggcu | 2.55 | 0.00 |
| 38 | hsa-miR-3195 | cgcgccgggcccggguu | 2.54 | 0.00 |
| 39 | hsa-miR-3196 | cggggcggcaggggccuc | 2.51 | 0.00 |
| 40 | hsa-miR-6724-5p | cugggcccgcggcgggcgugggg | 2.51 | 0.01 |
| 41 | hsa-miR-1268a | cgggcguggugguggggg | 2.49 | 0.01 |
| 42 | hsa-miR-6786-5p | gcgguggggccggaggggcgu | 2.49 | 0.01 |
| 43 | hsa-miR-1229-5p | guggguaggguuugggggagagcg | 2.46 | 0.04 |
| 44 | hsa-miR-3135b | ggcuggagcgagugcaguggug | 2.43 | 0.00 |
| 45 | hsa-miR-6769b-5p | ugguggguggggaggagaagugc | 2.42 | 0.01 |
| 46 | hsa-miR-4669 | uguguccgggaaguggaggagg | 2.4 | 0.03 |
| 47 | hsa-miR-7847-3p | cguggaggacgaggaggaggc | 2.35 | 0.05 |
| 48 | hsa-miR-4442 | gccggacaagagggagg | 2.34 | 0.04 |
| 49 | hsa-miR-1273g-3p | accacugcacuccagccugag | 2.32 | 0.04 |
| 50 | hsa-miR-718 | cuuccgccccgccgggcgucg | 2.32 | 0.00 |
| 51 | hsa-miR-6891-5p | uaaggagggggaugagggg | 2.3 | 0.04 |
| 52 | hsa-miR-30d-5p | uguaaacauccccgacuggaag | 2.28 | 0.02 |
| 53 | hsa-miR-3162-5p | uuagggaguagaaggguggggag | 2.22 | 0.02 |
| 54 | hsa-miR-6794-5p | cagggggacugggggugagc | 2.22 | 0.03 |
| 55 | hsa-miR-6879-5p | cagggcagggaaggugggagag | 2.22 | 0.01 |
| 56 | hsa-miR-7108-5p | guguggccggcaggcgggugg | 2.22 | 0.01 |
| 57 | hsa-miR-2861 | ggggccuggcggugggcgg | 2.19 | 0.03 |
| 58 | hsa-miR-5100 | uucagaucccagcggugccucu | 2.19 | 0.03 |
| 59 | hsa-miR-423-5p | ugaggggcagagagcgagacuuu | 2.14 | 0.00 |
| 60 | hsa-miR-320a | aaaagcuggguugagagggcga | 2.07 | 0.01 |
| 61 | hsa-miR-126-3p | ucguaccgugaguaauaaugcg | 2.06 | 0.02 |
| 62 | hsa-miR-92a-3p | uauugcacuugucccggccugu | 2.06 | 0.03 |
| 63 | hsa-miR-29a-3p | uagcaccaucugaaaucgguua | 2.04 | 0.00 |
| 64 | hsa-miR-20b-5p | caaagugcucauagugcagguag | 2.03 | 0.04 |
| 65 | hsa-miR-320d | aaaagcuggguugagagga | 2.02 | 0.00 |
| 66 | hsa-miR-4323 | cagccccacagccucaga | 0.47 | 0.02 |
| 67 | hsa-miR-6819-3p | aagccucuguccccaccccag | 0.46 | 0.01 |
| 68 | hsa-miR-4769-3p | ucugccauccucccuccccuac | 0.37 | 0.01 |
| 69 | hsa-miR-6870-3p | gcucauccccaucuccuuucag | 0.35 | 0.01 |
| 70 | hsa-miR-940 | aaggcagggcccccgcucccc | 0.32 | 0.01 |
| 71 | hsa-miR-1281 | ucgccuccuccucuccc | 0.25 | 0.00 |
| 72 | hsa-miR-1825 | uccagugcccuccucucc | 0.25 | 0.01 |
| 73 | hsa-miR-6760-3p | acacuguccccuucuccccag | 0.23 | 0.01 |
| 74 | hsa-miR-1238-3p | cuuccucgucugucugcccc | 0.22 | 0.01 |
| 75 | hsa-miR-191-3p | gcugcgcuuggauuucgucccc | 0.22 | 0.00 |
| 76 | hsa-miR-4313 | agcccccuggccccaaaccc | 0.22 | 0.02 |
| 77 | hsa-miR-4665-3p | cucggccgcggcgcguagcccccgcc | 0.22 | 0.00 |
| 78 | hsa-miR-6813-3p | aaccuuggccccucuccccag | 0.22 | 0.01 |
| 79 | hsa-miR-4725-5p | agacccugcagccuucccacc | 0.21 | 0.01 |
| 80 | hsa-miR-6800-3p | caccucuccuggcaucgcccc | 0.2 | 0.01 |
| 81 | hsa-miR-6861-3p | uggaccucuccuccccag | 0.2 | 0.04 |
| 82 | hsa-miR-6069 | gggcuagggccugcugccccc | 0.19 | 0.00 |
| 83 | hsa-miR-6508-5p | ucuagaaaugcaugacccacc | 0.19 | 0.00 |
| 84 | hsa-miR-1304-3p | ucucacuguagccucgaacccc | 0.18 | 0.00 |
| 85 | hsa-miR-1228-3p | ucacaccugccucgcccccc | 0.17 | 0.00 |
| 86 | hsa-miR-6515-3p | ucucuucaucuaccccccag | 0.15 | 0.01 |
| 87 | hsa-miR-6851-3p | uggcccuuuguaccccuccag | 0.14 | 0.00 |
| 88 | hsa-miR-6797-3p | ugcaugacccuucccuccccac | 0.13 | 0.00 |
| 89 | hsa-miR-1234-3p | ucggccugaccacccaccccac | 0.12 | 0.00 |
| 90 | hsa-miR-4433a-5p | cgucccaccccccacuccugu | 0.12 | 0.00 |
| 91 | hsa-miR-6737-3p | ucugugcuucaccccuacccag | 0.12 | 0.00 |
| 92 | hsa-miR-6889-3p | ucugugccccuacuucccag | 0.11 | 0.01 |
| 93 | hsa-miR-3162-3p | ucccuaccccuccacucccca | 0.09 | 0.00 |
| 94 | hsa-miR-4649-3p | ucugaggccugccucucccca | 0.09 | 0.00 |
