# Supplementary material for: Expression profile of plasma microRNAs and their roles in diagnosis of mild to severe traumatic brain injury
Source: PLoS One. 2018 Sep 18;13(9):e0204051. doi: 10.1371/journal.pone.0204051 (PMC6143266; doi:10.1371/journal.pone.0204051)
Supplement: S2 Table — (DOCX) [file pone.0204051.s002.docx]

**Table S2** MiRNAs altered in plasma samples of Moderate TBI compared with HV.

| **No.** | **miRNA** | **Mature sequence** | **Fold change** | **p-value** |
| --- | --- | --- | --- | --- |
| 1 | hsa-miR-4788 | uuacggaccagcuaagggaggc | 5.04 | 0.03 |
| 2 | hsa-miR-6867-5p | uguguguguagaggaagaaggga | 4.05 | 0.01 |
| 3 | hsa-miR-4800-5p | aguggaccgaggaaggaagga | 3.93 | 0.00 |
| 4 | hsa-miR-4778-5p | aauucuguaaaggaagaagagg | 3.86 | 0.01 |
| 5 | hsa-miR-3138 | uguggacagugagguagagggagu | 3.55 | 0.00 |
| 6 | hsa-miR-6879-5p | cagggcagggaaggugggagag | 3.45 | 0.03 |
| 7 | hsa-miR-3141 | gagggcggguggaggagga | 3.37 | 0.00 |
| 8 | hsa-miR-4442 | gccggacaagagggagg | 3.29 | 0.04 |
| 9 | hsa-miR-3195 | cgcgccgggcccggguu | 3.17 | 0.01 |
| 10 | hsa-miR-4739 | aagggaggaggagcggaggggcccu | 3.07 | 0.02 |
| 11 | hsa-miR-762 | ggggcuggggccggggccgagc | 2.96 | 0.01 |
| 12 | hsa-miR-4644 | uggagagagaaaagagacagaag | 2.95 | 0.01 |
| 13 | hsa-miR-483-5p | aagacgggaggaaagaagggag | 2.91 | 0.03 |
| 14 | hsa-miR-6831-5p | uagguagagugugaggaggagguc | 2.86 | 0.01 |
| 15 | hsa-miR-4298 | cugggacaggaggaggaggcag | 2.79 | 0.01 |
| 16 | hsa-miR-6769b-5p | ugguggguggggaggagaagugc | 2.76 | 0.03 |
| 17 | hsa-miR-7847-3p | cguggaggacgaggaggaggc | 2.69 | 0.04 |
| 18 | hsa-miR-1268a | cgggcguggugguggggg | 2.67 | 0.01 |
| 19 | hsa-miR-3665 | agcaggugcggggcggcg | 2.66 | 0.04 |
| 20 | hsa-miR-6833-5p | guguggaagaugggaggagaaa | 2.58 | 0.00 |
| 21 | hsa-miR-6510-5p | cagcaggggagagagaggaguc | 2.49 | 0.05 |
| 22 | hsa-miR-3162-5p | uuagggaguagaaggguggggag | 2.47 | 0.05 |
| 23 | hsa-miR-4634 | cggcgcgaccggcccgggg | 2.46 | 0.04 |
| 24 | hsa-miR-6724-5p | cugggcccgcggcgggcgugggg | 2.43 | 0.03 |
| 25 | hsa-miR-4669 | uguguccgggaaguggaggagg | 2.4 | 0.05 |
| 26 | hsa-miR-130a-3p | cagugcaauguuaaaagggcau | 2.34 | 0.03 |
| 27 | hsa-miR-24-3p | uggcucaguucagcaggaacag | 2.31 | 0.01 |
| 28 | hsa-miR-328-5p | gggggggcaggaggggcucaggg | 2.3 | 0.02 |
| 29 | hsa-miR-6740-5p | aguuugggauggagagaggaga | 2.26 | 0.03 |
| 30 | hsa-miR-6794-5p | cagggggacugggggugagc | 2.23 | 0.01 |
| 31 | hsa-miR-7107-5p | ucggccuggggaggaggaaggg | 2.14 | 0.04 |
| 32 | hsa-miR-2861 | ggggccuggcggugggcgg | 2.07 | 0.03 |
| 33 | hsa-miR-3135b | ggcuggagcgagugcaguggug | 2.05 | 0.00 |
| 34 | hsa-miR-4769-3p | ucugccauccucccuccccuac | 0.44 | 0.03 |
| 35 | hsa-miR-6870-3p | gcucauccccaucuccuuucag | 0.43 | 0.01 |
| 36 | hsa-miR-6819-3p | aagccucuguccccaccccag | 0.36 | 0.00 |
| 37 | hsa-miR-940 | aaggcagggcccccgcucccc | 0.32 | 0.01 |
| 38 | hsa-miR-1238-3p | cuuccucgucugucugcccc | 0.3 | 0.02 |
| 39 | hsa-miR-6760-3p | acacuguccccuucuccccag | 0.3 | 0.02 |
| 40 | hsa-miR-6813-3p | aaccuuggccccucuccccag | 0.3 | 0.02 |
| 41 | hsa-miR-1825 | uccagugcccuccucucc | 0.29 | 0.01 |
| 42 | hsa-miR-191-3p | gcugcgcuuggauuucgucccc | 0.29 | 0.01 |
| 43 | hsa-miR-6508-5p | ucuagaaaugcaugacccacc | 0.29 | 0.01 |
| 44 | hsa-miR-6800-3p | caccucuccuggcaucgcccc | 0.27 | 0.02 |
| 45 | hsa-miR-1304-3p | ucucacuguagccucgaacccc | 0.26 | 0.00 |
| 46 | hsa-miR-4313 | agcccccuggccccaaaccc | 0.26 | 0.03 |
| 47 | hsa-miR-4725-5p | agacccugcagccuucccacc | 0.26 | 0.01 |
| 48 | hsa-miR-6069 | gggcuagggccugcugccccc | 0.25 | 0.00 |
| 49 | hsa-miR-1281 | ucgccuccuccucuccc | 0.24 | 0.00 |
| 50 | hsa-miR-4433a-5p | cgucccaccccccacuccugu | 0.23 | 0.01 |
| 51 | hsa-miR-6515-3p | ucucuucaucuaccccccag | 0.23 | 0.01 |
| 52 | hsa-miR-6737-3p | ucugugcuucaccccuacccag | 0.22 | 0.01 |
| 53 | hsa-miR-1228-3p | ucacaccugccucgcccccc | 0.21 | 0.00 |
| 54 | hsa-miR-4665-3p | cucggccgcggcgcguagcccccgcc | 0.21 | 0.00 |
| 55 | hsa-miR-6851-3p | uggcccuuuguaccccuccag | 0.21 | 0.01 |
| 56 | hsa-miR-6797-3p | ugcaugacccuucccuccccac | 0.2 | 0.01 |
| 57 | hsa-miR-1234-3p | ucggccugaccacccaccccac | 0.18 | 0.01 |
| 58 | hsa-miR-4649-3p | ucugaggccugccucucccca | 0.18 | 0.01 |
| 59 | hsa-miR-6889-3p | ucugugccccuacuucccag | 0.18 | 0.02 |
| 60 | hsa-miR-3162-3p | ucccuaccccuccacucccca | 0.17 | 0.01 |
