# Supplementary material for: Expression profile of plasma microRNAs and their roles in diagnosis of mild to severe traumatic brain injury
Source: PLoS One. 2018 Sep 18;13(9):e0204051. doi: 10.1371/journal.pone.0204051 (PMC6143266; doi:10.1371/journal.pone.0204051)
Supplement: S3 Table — (DOCX) [file pone.0204051.s003.docx]

**Table S3** MiRNAs altered in plasma samples of Severe TBI compared with HV.

| **No.** | **miRNA** | **Mature sequence** | **Fold change** | **p-value** |
| --- | --- | --- | --- | --- |
| 1 | hsa-miR-223-3p | ugucaguuugucaaauacccca | 5.77 | 0.04 |
| 2 | hsa-miR-6867-5p | uguguguguagaggaagaaggga | 4.32 | 0.01 |
| 3 | hsa-miR-7107-5p | ucggccuggggaggaggaaggg | 3.63 | 0.01 |
| 4 | hsa-miR-3665 | agcaggugcggggcggcg | 3.56 | 0.02 |
| 5 | hsa-miR-4788 | uuacggaccagcuaagggaggc | 3.43 | 0.03 |
| 6 | hsa-miR-3195 | cgcgccgggcccggguu | 3.23 | 0.01 |
| 7 | hsa-miR-483-5p | aagacgggaggaaagaagggag | 3.19 | 0.02 |
| 8 | hsa-miR-197-5p | cggguagagagggcagugggagg | 3.02 | 0.02 |
| 9 | hsa-miR-4669 | uguguccgggaaguggaggagg | 2.75 | 0.04 |
| 10 | hsa-miR-6510-5p | cagcaggggagagagaggaguc | 2.65 | 0.04 |
| 11 | hsa-miR-6727-5p | cucggggcaggcggcugggagcg | 2.62 | 0.04 |
| 12 | hsa-miR-762 | ggggcuggggccggggccgagc | 2.39 | 0.01 |
| 13 | hsa-miR-328-5p | gggggggcaggaggggcucaggg | 2.33 | 0.02 |
| 14 | hsa-miR-6740-5p | aguuugggauggagagaggaga | 2.33 | 0.02 |
| 15 | hsa-miR-4530 | cccagcaggacgggagcg | 2.16 | 0.05 |
| 16 | hsa-miR-2861 | ggggccuggcggugggcgg | 2.13 | 0.03 |
| 17 | hsa-miR-1825 | uccagugcccuccucucc | 0.4 | 0.05 |
| 18 | hsa-miR-1281 | ucgccuccuccucuccc | 0.39 | 0.02 |
| 19 | hsa-miR-1304-3p | ucucacuguagccucgaacccc | 0.38 | 0.03 |
| 20 | hsa-miR-4725-5p | agacccugcagccuucccacc | 0.37 | 0.04 |
| 21 | hsa-miR-940 | aaggcagggcccccgcucccc | 0.37 | 0.02 |
| 22 | hsa-miR-4665-3p | cucggccgcggcgcguagcccccgcc | 0.33 | 0.02 |
